# Patient Factors Associated With Delays in Obtaining Cancer Care in Botswana

**DOI:** 10.1200/JGO.18.00088

**Published:** 2018-09-10

**Authors:** Rohini K. Bhatia, Sarah Rayne, William Rate, Lame Bakwenabatsile, Barati Monare, Chidinma Anakwenze, Preet Dhillon, Mohan Narasimhamurthy, Scott Dryden-Peterson, Surbhi Grover

**Affiliations:** **Rohini K. Bhatia**, University of Rochester School of Medicine and Dentistry, Rochester, NY; **Sarah Rayne**, University of the Witwatersrand, Johannesburg, South Africa; **William Rate**, Georgetown University School of Medicine, Washington, DC; **Lame Bakwenabatsile** and **Barati Monare**, Botswana-University of Pennsylvania Partnership; **Mohan Narasimhamurthy**, University of Botswana; **Surbhi Grover**, Princess Marina Hospital, Gaborone, Botswana; **Surbhi Grover,** University of Pennsylvania, Philadelphia, PA; **Chidinma Anakwenze**, University of Texas Health Science Center at Houston, McGovern Medical School, Houston, TX; **Preet Dhillon**, Public Health Foundation of India, Gurgaon, India; **Scott Dryden-Peterson**, Brigham and Women’s Hospital and Botswana Harvard AIDS Institute, Harvard TH Chan School of Public Health, Boston, MA.

## Abstract

**Purpose:**

Delays in diagnosis and treatment of cancers can lead to poor survival. These delays represent a multifaceted problem attributable to patient, provider, and systemic factors. We aim to quantify intervals from symptom onset to treatment start among patients with cancer in Botswana and to understand potential risk factors for delay.

**Patients and Methods:**

From December 2015 to January 2017, we surveyed patients seen in an oncology clinic in Botswana. We calculated proportions of patients who experienced delays in appraisal (between detecting symptoms and perceiving a reason to discuss them with provider, defined as > 1 month), help seeking (between discussing symptoms and first consultation with provider, defined as > 1 month), diagnosis (between first consultation and receiving a diagnosis, defined as > 3 months), and treatment (between diagnosis and starting treatment, defined as > 3 months).

**Results:**

Among 214 patients with cancer who completed the survey, median age at diagnosis was 46 years, and the most common cancer was cancer of the cervix (42.2%). Eighty-one percent of patients were women, 60.7% were HIV infected, and 56.6% presented with advanced cancer (stage III or IV). Twenty-six percent of patients experienced delays in appraisal, 35.5% experienced delays help seeking, 63.1% experienced delays in diagnosis, and 50.4% experienced delays in treatment. Patient income, education, and age were not associated with delays. In univariable analysis, patients living with larger families were less likely to experience a help-seeking delay (odds ratio [OR], 0.31; *P* = .03), women and patients with perceived very serious symptoms were less likely to experience an appraisal delay (OR, 0.45; *P* = .032 and OR, 0.14; *P* = .02, respectively).

**Conclusion:**

Nearly all patients surveyed experienced a delay in obtaining cancer care. In a setting where care is provided without charge, cancer type and male sex were more important predictors of delays than socioeconomic factors.

## INTRODUCTION

Cancer disproportionately affects populations in low- and middle-income countries. In 2012, there were 14.1 million new patients diagnosed with cancer worldwide, and this number is projected to increase to 21 million in 2030.^1,2^ Despite recent advances, disparities in cancer survival continue to persist. For example, the probability of a woman dying from cancer is two times higher in Africa compared with Europe.^2^

Botswana is a middle-income country in sub-Saharan Africa with a population of approximately 2.2 million. Oncology services are available without cost to Botswana citizens at Princess Marina Hospital (PMH), a government and referral hospital in the capital of Gaborone, and Nyangabgwe Referral Hospital in Francistown, 400 km from Gaborone. At the time of this study, services were also available in public hospitals in Maun and Serowe, cities in the north of the country. The only radiation center in the country is located at a private facility in Gaborone. The cost of radiation for patients referred from government hospitals is fully supported with public funds. PMH is the largest facility, consistently has an oncologist on staff, and is in close proximity to the radiation center; thus, it sees the majority of nationwide reported patients with cancer.^3^

Recent changes in lifestyle in Botswana have increased carcinogenic risk factors, and an HIV prevalence of 22% among adults^4^ underlies a high incidence of immune- and viral-mediated cancers.^5,6^ In the early 2000s, a successful nationwide antiretroviral campaign was initiated,^7^ introducing longer life expectancy and thus increasing the natural risk for acquiring cancer.^8^

**Fig 1:**
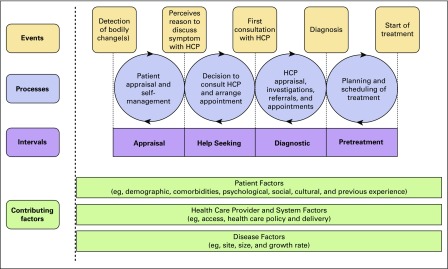
Walter et al^16^ Modified Andersen Model (2012) defining the different treatment periods along the cancer care spectrum. HCP, health care provider.

It is well known that cancers detected at advanced stages have a higher mortality rate than those that are localized and present earlier.^9,10^ Although there are a multitude of reasons for cancer to present at an advanced stage, delays in presentation, diagnosis, and treatment are critical considerations that have the potential to be targeted by public health initiatives.^11,12^ Literature suggests that the time between an individual recognizing symptoms and presenting to a health care facility may be influenced by severity or awareness of symptoms, perception of personal risk, and physical or social barriers to accessing care.^13-15^ Walter et al^16^ recently proposed a theoretical framework, expanding the Andersen Model of Total Patient Delay, to create a consensus on definitions and treatment periods. In this model, appraisal is defined as the time between detecting symptoms and perceiving a reason to discuss them with someone; help seeking is the time between deciding to discuss symptoms and first consultation with a health care provider (HCP); diagnostic is defined as the time between a patient’s first consultation with an HCP and diagnosis; and treatment is the interval between diagnosis and the start of treatment (Fig 1).^16^

Previous work regarding delays in Botswana concluded that the median time between symptom onset and treatment was 13 months compared with 3 to 5 months in more developed countries; the presence of HIV did not affect this time despite patients with HIV having more frequent contact with the health system.^17-19^ We hope to better understand the sociodemographic factors, clinical factors, and beliefs associated with delayed cancer presentation in Botswana. By applying a theoretical framework, we can highlight potential points of intervention to improve delays along the care spectrum.

## PATIENTS AND METHODS

### Sampling and Data Collection

From December 2015 to January 2017, patients seen at the PMH oncology clinic in Gaborone were recruited to complete a questionnaire. A convenience sample was recruited from PMH with the following eligibility criteria: patients > 18 years of age with a pathologically confirmed new diagnosis of cancer. Consenting patients were asked to complete a questionnaire, which has been previously described.^20^ The questionnaire was developed and adapted for Botswana and read to patients by a researcher trained in its administration.

### Covariate Descriptions

Covariates under five different categories were measured, which included sociodemographics; prediagnosis events; diagnosis; family support; and fears, knowledge, and stigma.

### Outcomes

A reference to treatment periods in this article refers to the appraisal, help-seeking, diagnosis, and treatment stages on the cancer care spectrum. Each treatment period was assessed as follows. Appraisal was assessed with the following question: “How long before you told anyone else or sought help did you notice there might be a problem?” Health seeking was assessed with the following question: “After that, how long was it before you got help from a hospital or clinic?” Diagnosis was assessed with the following question: “From the time you went to see a doctor, how long was it until you got a confirmed diagnosis of cancer?” Treatment was evaluated using the diagnosis date and treatment start date as documented in the PMH medical record system. A delay in these categories was defined according to previous literature,^13,14,21,22^ clinical expertise, and knowledge about in-country resources and standards.^17,23^ Participants were asked to choose from four different time intervals that were then collapsed into two variables (ie, ≤ or > 3 months) to define presence of delay. A delay in appraisal or help-seeking behavior was defined as **>** 1 month. A delay in diagnosis or treatment was defined as **>** 3 months.

### Data Management and Ethics

Responses to a four-point scale of statements of fears, knowledge, and beliefs (from strongly agree to strongly disagree) were collapsed into agree or disagree. Additional clinical information, including treatment and cancer stage, was collected for each patient from patient cards and the electronic medical system at the time of survey administration.

Patient data were de-identified, and study data were collected and managed using Research Electronic Data Capture tools hosted at the University of Pennsylvania School of Medicine (Philadelphia, PA). The study received ethical approval from the institutional review boards of the University of Pennsylvania and PMH and the Health Research Development Committee at the Botswana Ministry of Health.

### Analysis

Demographic and clinical characteristics were described using proportions and stratified by HIV status, sex, and presence of delays in each treatment period. χ^2^ analysis was used to compare patients in different groups. *P* < .05 was considered statistically significant. Univariable (UVA) logistic regression was used to determine the presence of significant factors in predicting delay. Factors analyzed using UVA were sex, age, relationship status, religious beliefs, education, number of family members, comorbidities, distance from hospital, time to hospital, symptom severity, use of traditional physician, cancer diagnosis site, and cancer stage.

A Kruskal-Wallis *H* test was conducted to determine whether fears, knowledge, and beliefs were different among those who experienced a delay and those who did not. A post hoc analysis was then performed to determine probability of experiencing a delay given agreement to a fear, attitude, or belief statement. All statistical analyses were done in STATA software (Version 13.1, STATA, College Station, TX).

## RESULTS

### Sociodemographics

A convenience sample of 214 patients who presented for oncology care at PMH consented to and completed the survey. Table 1 lists demographic characteristics of all patients by HIV status and sex. Participants were 21 to 95 years of age, with a median age of 46 years (interquartile range, 39 to 55 years). Similar to the characteristics of patients with cancer in Botswana, 174 patients (81.3%) were women, and 128 (60.7%) were HIV infected. Primary schooling (from first to seventh standard) was completed by one third of patients. Patients lived a median of 72 km and a mean of 182 km away from PMH, and 84 patients (39.8%) noted that it took 1 to 4 hours to travel to the hospital. Patients infected with HIV were more likely to be younger (*P* < .001), employed (*P* = .032), and single (*P* < .001) than patients not infected. HIV status was not associated with cancer stage.

**Table 1:**
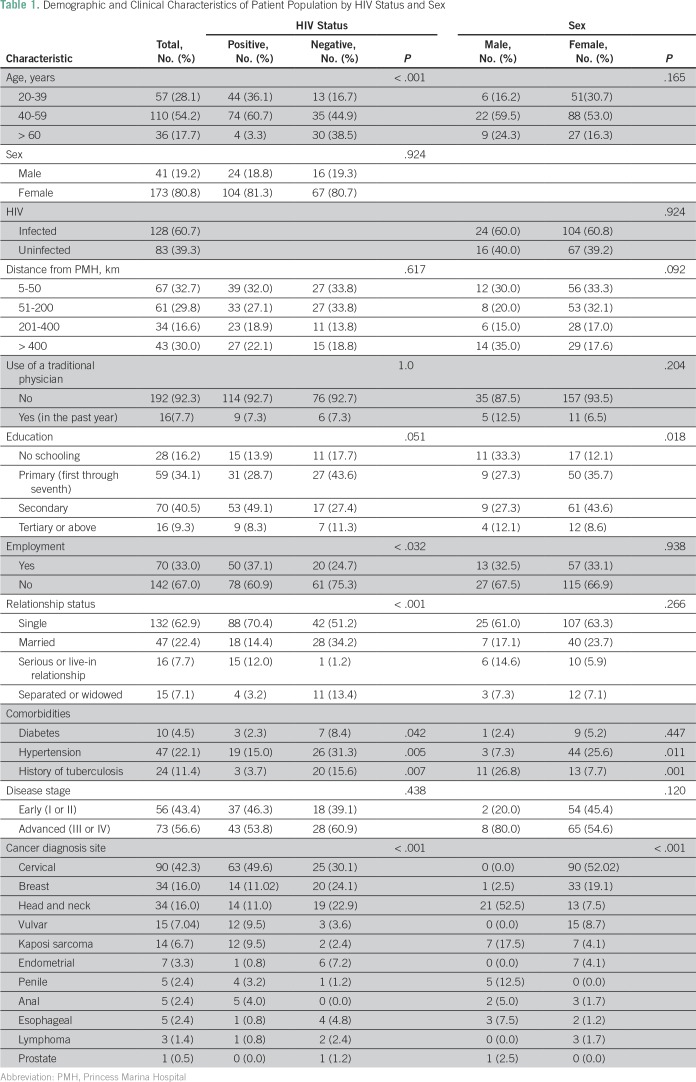
Demographic and Clinical Characteristics of Patient Population by HIV Status and Sex

### Clinical Features

Table 1 lists the clinical features for all patients and by HIV status and sex. Among these patients the most common site of cancer diagnosis was the cervix (n = 90, 42.2%), followed by the head and neck (n = 33, 15.5%). Across all patients with cancer, 73 patients (56.6%) presented with advanced stage (stage III or IV). Comorbid illness (HIV, diabetes, hypertension, and/or tuberculosis) was present in 74.3% of patients. The most common comorbidity was HIV (n = 128, 60.6%), followed by hypertension (n = 47, 22.1%).

One hundred thirty patients (60.8%) reported symptom discomfort as the reasons for going to a physician; 73 patients (34%) reported increasing worry. Most patients did not try outside remedies before seeing an allopathic physician (n = 159, 74.3%); however, among the 49 patients who did, 32 patients (65%) visited a healing church and 11 patients (22%) visited a traditional healer.

### Delays

Table 2 lists demographic and clinical features by delays in different treatment periods. and Table 3 lists the results of UVA of significant factors associated with these delays. The most common time interval for appraisal was < 1 week (n = 100, 46%). A quarter of patients (n = 55, 25.7%) experienced an appraisal delay as defined as **>** 1 month. In χ^2^ analysis, as shown in Table 2, male sex was a significant factor contributing to delay (delays in men *v* women, 39% *v* 22%, respectively; *P* = .022). In addition, among those who viewed their symptoms as very serious, only 19 patients (25%) experienced a delay (*P* = .02). UVA of factors associated with delay, as listed in Table 3, revealed that patients with Kaposi sarcoma (n = 14, 6.6%) and penile cancer (n = 5, 2.4%) were more likely to experience an appraisal delay (odds ratio [OR], 9.77; *P* < .001 and OR, 8.14; *P* = .029, respectively); patients with cervical or breast cancer were significantly less likely to experience an appraisal delay when compared with all other cancer types (OR, 0.372; *P* = .002).

**Table 2:**
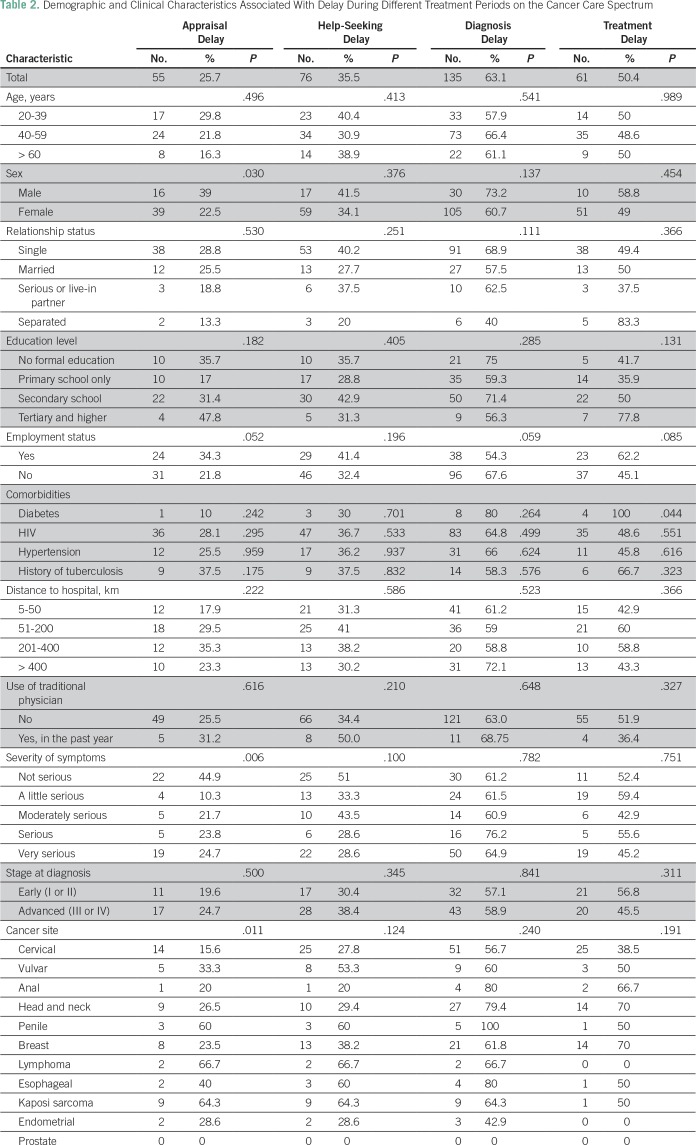
Demographic and Clinical Characteristics Associated With Delay During Different Treatment Periods on the Cancer Care Spectrum

**Table 3:**
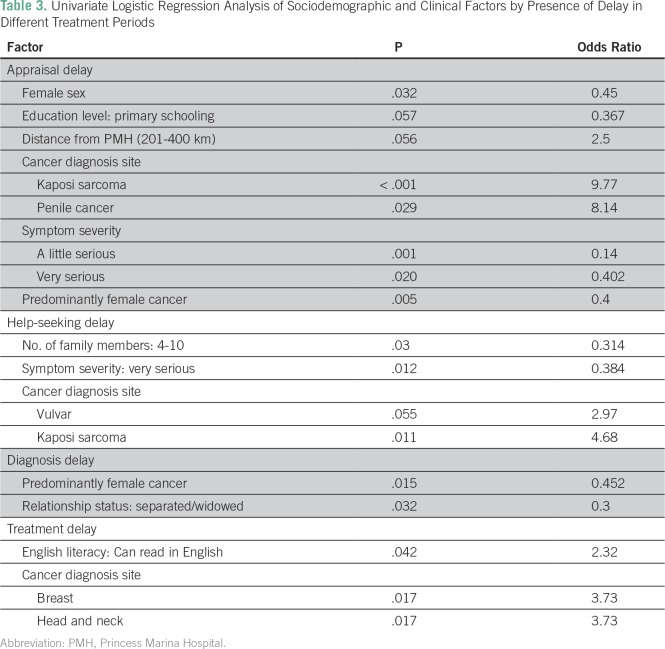
Univariate Logistic Regression Analysis of Sociodemographic and Clinical Factors by Presence of Delay in Different Treatment Periods

The most common time interval for seeking help was between 1 week and 1 month (n = 72, 33.6%). More than one third of patients (n = 76, 5.5%) experienced a help-seeking delay (> 1 month) for their symptom. Those who defined their symptoms as very serious were less likely to experience a help-seeking delay (OR, 0.402; *P* = .02) in UVA (Table 3). A greater number of family members was protective against delay (four to 10 members: OR, 0.314; *P* = .003; Table 3).

The most common time interval for diagnosis was > 6 months (n = 75, 35.01%). One hundred thirty-five patients (63.1%) experienced a diagnosis delay, defined as > 3 months (Table 2). Patients with predominantly female cancers were significantly less likely to experience this delay (OR, 0.4; *P* = .005; Table 3).

One hundred twenty-one of 214 patients had begun treatment as per patient records. The mean number of days between diagnosis and treatment was 155, and the median was 91 days (interquartile range, 63 to 238 days). Overall, 61 patients (50.4%) had a treatment delay, as defined as > 3 months from date of diagnosis to their start of treatment. In logistic regression, patients with head and neck cancer (OR, 3.7; *P* = .017) and breast cancer (OR, 3.7; *P* = .017) were more likely to experience a treatment delay. Across all points of delay, distance, time to hospital, and use of a traditional physician were not statistically significant contributors to delay in our analysis, as shown in Table 2.

### Fears, Knowledge, and Beliefs

One hundred sixty seven patients (80%) believed that faith in God alone would heal them, 135 patients (65.2%) believed that if someone got cancer it was part of God’s plan, and 142 patients (68.6%) believed that people have been cured of cancer through prayer and faith alone. Almost all patients believed that there is a treatment for cancer (n = 195, 94.2%) and that it is survivable (n = 195, 94.2%). The most common fear among patients was of death and (n = 30, 14.1%) and of missing appointments or treatment because of a lack of money (n = 31, 14.6%). The most commonly held belief was that elderly individuals get cancer (n = 98, 47.3%); forty two patients (20%) believed that contraception could cause cancer, and a similar proportion believed bad genes (n = 48, 22.9%) and stress or anxiety (n = 38, 18.4%) could cause cancer.

Table 4 lists the results of the Kruskal-Wallis *H* test, which determines statistically significant differences in agreeing with belief statements among those who did and did not experience a delay. Patients who believed that cancer is part of God’s plan demonstrated a protective effect against an appraisal delay (OR, 0.272, *P* < .001) and help-seeking delay (OR, 0.429; *P* = .0013).

**Table 4:**
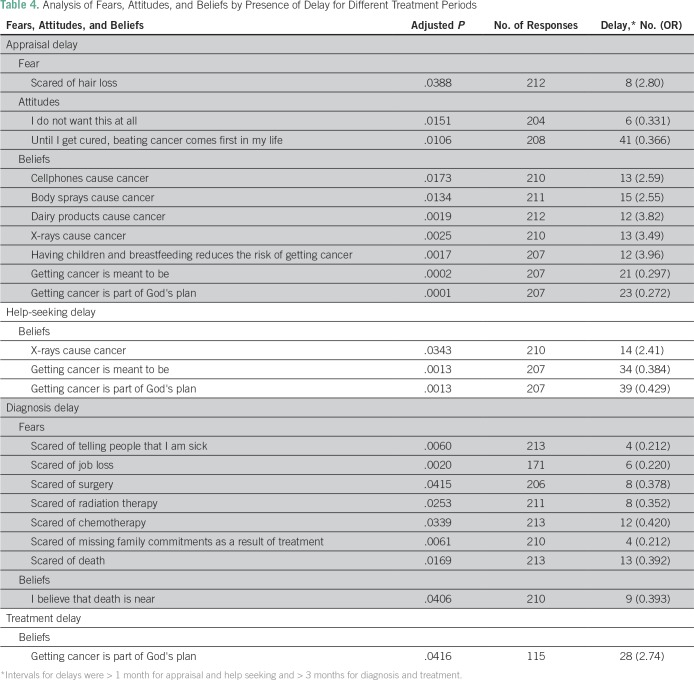
Analysis of Fears, Attitudes, and Beliefs by Presence of Delay for Different Treatment Periods

## DISCUSSION

In Botswana, delay is evident across all treatment periods and multifactorial in nature, influenced by symptoms, sex, and type of cancer. This survey identified risk factors and beliefs associated with different points of delay using a theoretical framework.^16^

Many studies on patient delay in cancer presentation have been conducted in Western Europe, the United States, and Australia.^11^ In addition, there is a large body of literature concerning delays in presentation for specific cancers, specifically breast cancer.^14,21,24-28^ There is a dearth of literature concerning overall cancer delay in low- and middle-income countries, although recently, studies from India and Botswana have emerged.^17,29,30^

Our outcome measures of delay were defined using a combination of feedback from local HCPs and previous literature. Pack and Gallo^31^ defined cancer delay as a dichotomous variable in 1938, and their definition of > 3 months between symptoms and presentation has now been used in various studies.^13,32-34^ Still others have adhered to a delay interval of 1 month,^35^ 30 days,^36^ or 8 weeks,^37^ or based on the spread of the data.

The most common appraisal interval across all cancer types in this study was less than a week. Delay data from across Africa of appraisal in breast cancer range from 2 weeks to 11 years after self-noticing an abnormality to presenting to an HCP.^38^ Consistent with previous studies, our data show that men were more likely to have a delay in appraisal of cancer symptoms than women.^11,17^ Men may view discussing symptoms as not masculine, citing that women are more likely to seek help because of frequent contact with the health system.^11^ Qualitative research into men’s help-seeking beliefs after recognizing a symptom in Botswana is needed to provide more insight.

Symptom severity and underlying fear and embarrassment have been documented to contribute to symptom appraisal.^11^ Walter et al^16^ noted that the most important factor for determining appraisal delay was the nature of the patient’s symptoms, including the misattribution of a serious symptom to a comorbid illness or previously benign condition, especially in patients with ovarian cancer. Our data are consistent with previous studies noting that symptom severity can affect time to seek additional resources. Furthermore, it is known that recognition of a specific symptom, such as a lump for breast cancer, is associated with reduced delay compared with those experiencing vague symptoms.^12^

Patients with a greater number of family members were less likely to experience a help-seeking delay. Previous literature has noted that men in relationships and those who receive partner support were less likely to experience patient-related delay. Women tend to have larger social networks and rely on these for emotional support and advice, which also reduces delay.^39^ Understanding what types of social networks may prompt an individual to see a physician may encourage programs that prompt spousal conversation or family discussions about cancer screening in Botswana.

Diagnosis delay was most prevalent in our study, consistent with the findings of Brown et al.^17^ A greater number of patients experienced diagnosis and treatment delays in our study compared with appraisal and help-seeking delays. This underscores the need to address system-related (*v* patient-related) factors. In our study, patients with female cancers were less likely to experience a diagnostic delay. Recently, there has been a concerted effort to improve resources for gynecologic cancer screening, diagnosis, and treatment in Botswana.^40^ This may explain the fewer delays for female cancers and also serve as a model for other cancer sites.^17^

Among the fears, knowledge, and beliefs assessed in this study, > 65% of patients surveyed expressed that cancer could be cured through prayer and faith or that getting cancer was part of God’s plan. In a previous study in which African American women were surveyed on their beliefs surrounding breast cancer diagnosis and treatment, women who only shared knowledge of their symptoms with God were more likely to delay seeking medical care.^41^ In a study of Latina women’s cultural beliefs in relation to cancer, a common belief held was that faith in God can prevent breast cancer. The authors found that women with a higher number of these cultural beliefs were more likely to have prolonged delays.^42^ These findings suggest that engaging religious leaders or traditional healers may be an important dissemination tool for public health information.

The most common fear among patients was of missing appointments or treatment because of a lack of money. Although the Botswana government covers the cost of treatment of all citizens, certain items are not included in the coverage, such as medications not available in the public sector, travel, lodging, or loss of income from missed time at work. Additional investigations into the financial burdens patients encounter are necessary to create a supportive treatment pathway.

There are several limitations to this study, including the small sample size caused by logistical difficulties in accrual. In addition, cancer subtypes have different presenting symptoms, follow-up, and treatment plans. This study was an overview of the general oncologic patient pathway; however, cancer type–specific studies can better highlight potential intervention steps. In using the Aarhus Statement Working Group findings as a framework, we note that our retrospective study is subject to recall and social desirability bias because it required patients to remember times of first noticing a symptom, telling someone about the symptom, and presenting to a physician.^43^ Furthermore, we acquired categorical (*v* continuous numerical) time data for delays. Many of the cofactors we analyzed were patient-related factors, and we recognize the need to follow-up with future studies focused on systematic and economic factors associated with delay. Finally, study participants were those that presented for care, and there are likely many who do not present for care. Although beyond the scope of this study, future investigations into presumed patients with cancer at the community level can be undertaken to understand factors associated with patient choice to receive or not receive treatment after diagnosis. We ascertained treatment information on just more than half of our sample population, and thus, we are limited in our generalization of conclusions of the pretreatment interval.

Nevertheless, this study contributes to growing literature regarding delayed presentation with cancer symptoms to health care facilities in Botswana. The identification of risk factors, including sex, type of cancer, and education, can provide important targets of potential public health intervention programs. Additional investigations into how using professional education to assist HCPs in recognizing symptoms and referring to appropriate specialists to reduce diagnostic delays are needed for specific cancers. In addition, promoting dialogue of cancer causes and symptoms through use of both familial and religious networks may be an effective strategy to disseminate knowledge. In a setting where care is provided without charge, cancer type and male sex were more important predictors of delays than socioeconomic factors. Identifying factors that contribute to longer intervals is an important step in improving patients’ experience with the health care system and reducing time to care and treatment.
